# Supplementary material for: Effect of a Telephone-Based Lifestyle Intervention on Weight, Body Composition, and Metabolic Biomarkers in Rural Ohio: Results from a Randomized Pilot Study
Source: Nutrients. 2023 Sep 15;15(18):3998. doi: 10.3390/nu15183998 (PMC10538144; doi:10.3390/nu15183998)
Supplement: Supplementary file 1 [file nutrients-15-03998-s001.zip › nutrients-2586961-supplementary.pdf]

Table S1. Baseline Characteristics between study completers vs. dropouts.

Characteristics	Completed the study n=33	Dropped out n=7	P
Age	49.1±10.0	49.4±5.6	0.94
BMI	35.4±5.85	41.5±2.8	0.01
Gender			0.45
Male	2 (6.1%)	1(14.3%)	
Female	31 (93.9%)	6(85.7%)	
